# Pressure mat analysis of naturally occurring lameness in young pigs after weaning

**DOI:** 10.1186/s12917-014-0193-8

**Published:** 2014-08-20

**Authors:** Ellen Meijer, Maarten Oosterlinck, Arie van Nes, Willem Back, Franz Josef van der Staay

**Affiliations:** 1 Department of Farm Animal Health, Faculty of Veterinary Medicine, Utrecht University, Yalelaan 7, Utrecht, NL-3584, CL, The Netherlands; 2 Department of Surgery and Anaesthesiology, Faculty of Veterinary Medicine, Ghent University, Merelbeke, B-9820, Belgium; 3 Department of Equine Sciences, Faculty of Veterinary Medicine, Utrecht University, Yalelaan 112-114, Utrecht, NL-3584, CM, The Netherlands

**Keywords:** Kinetics, Gait analysis, Porcine, Symmetry, Redistribution, Loading

## Abstract

**Background:**

Lameness is a common problem in modern swine husbandry. It causes welfare problems in affected pigs as well as financial problems for farmers. To minimize these negative consequences of lameness, new treatment and prevention strategies need to be developed and validated using objective and quantitative measurement techniques. An example of such a putative diagnostic tool is the use of a pressure mat. Pressure mats are able to provide both objective loading (kinetic) as well as objective movement (kinematic) information on pig locomotion.

In this study, pressure mat analysis was used to assess compensatory force redistribution in lame pigs; in particular a predefined set of four pressure mat parameters was evaluated for its use to objectively distinguish clinically lame from sound pigs. Kinetic data from 10 clinically lame and 10 healthy weaned piglets were collected. These data were analyzed to answer three research questions. Firstly the pattern of compensatory weight distribution in lame animals was studied using the asymmetry indices (ASI) for several combinations of limbs. Secondly, the correlation between total left-right asymmetry index and visual scores of lameness was assessed. Thirdly, by using receiver-operated curve (ROC) analysis, optimal cutoff values for these ASIs were then calculated to objectively detect lame pigs.

**Results:**

Lame animals generally showed a shift in loading towards their diagonal and contralateral limbs, resulting in a clear left-right asymmetry. The degree of lameness as graded by visual scoring correlated well with the total left-right ASIs. Lame pigs could be objectively distinguished from sound pigs based on clear cutoff points calculated by ROC analysis for the complete set of four evaluated parameters.

**Conclusions:**

The gait of lame pigs is asymmetric, due to the unloading of the affected limb and concomitant weight redistribution towards other limbs. This asymmetry objectively expressed as total left-right asymmetry, correlates well with the subjective visual lameness scoring and can be used to objectively distinguish lame from sound pigs. Pressure mat gait analysis of pigs, therefore, appears to be a promising and useful tool to objectively quantify and possibly early detect lameness in pigs.

## Background

Lameness in pigs is a common problem in modern swine husbandry, affecting up to 19% of finishing pigs [1]. It has negative consequences both from an animal welfare as well as from an economic point of view. Lameness seriously impairs welfare and may have an effect on all of Brambell’s “five freedoms” [2]. The economic impact of lameness is caused by lower productivity in lame animals, the cost of treating affected animals and the cost of premature culling of animals [3],[4]. The aforementioned welfare and economic issues can be minimized if veterinarians provide evidence-based advice on treating and preventing lameness. Detection of lameness needs to be sensitive and reliable, and treatment and prevention strategies need to be assessed using objective, repeatable methods to quantify lameness.

As already proven in other animals including man, pressure mats are a noninvasive and objective tool to quantitatively assess gait, providing kinetic and temporospatial data. Their use has increased lately as they seem to have some distinct advantages over other, ‘classical’ methods to analyze gait, like force plates and infrared high-speed camera systems. When using pressure mats, it is possible to measure consecutive, overlapping and even simultaneous footfalls, thus enabling the collection of several footfalls in one run. Moreover, an elaborate calibration setup that some other, aforementioned methods would require for a 3D kinetic and kinematic analysis is not needed. In addition, pressure plates with high sensor density provide information on the pressure distribution between different regions, for example between different claws within the foot.

Pressure mats have been used to study gait in several other quadruped species, such as horses [5], cattle [6], sheep [7], dogs [8], and cats [9]. Most of these studies focused on sound animals. Considerably less information, however, is available on the sensitivity and reliability of pressure mats to objectively distinguish lame from sound animals. Earlier studies in dogs and cattle indeed reported that it is possible to distinguish lame from sound individuals [8],[10]–[12]. However, hardly any information on the use of pressure mat analysis to detect lameness in pigs is available yet. Only one single study was identified that described lameness in sows using pressure mat analysis, but only peak vertical pressure symmetry was used to objectively quantify lameness [13].

Therefore, the objective of this study was to evaluate the ability of four kinetic pressure mat parameters (peak vertical force: PVF, load rate: LR, vertical impulse: VI, and peak vertical pressure: PVP) to distinguish clinically lame from sound weaned piglets.

We assessed compensatory weight redistribution by calculating the asymmetry-indices (ASI) for several combinations of limbs and for each of the pressure mat parameters. In order to compare this new method to the method that is currently used most often in lameness research in pigs (visual scoring), correlations of total left-right ASIs for the four pressure mat parameters with visual scores of lameness were assessed as well. We used receiver-operated characteristic (ROC) curve analysis to determine the optimal cutoff values of total left-right ASI to identify lame pigs.

## Results

Mean body mass was 9.5 ± 2.0 kg for the lame pigs and 9.1 ± 1.7 kg for the sound pigs and did not differ between groups (t(18) = 0.462, p = 0.650). Mean velocity was 1.3 ± 0.5 m/s in the sound pigs and 0.6 ± 0.4 m/s in the lame pigs. The velocity was lower in the lame pigs compared to the sound pigs (t(18) = 4.146, p = 0.001).

The visual gait score (0 = sound, 5 = non-weigh bearing lameness) in the lame pigs ranged from 2 to 4 with a mean of 3.0 ± 0.8.

Details of the gross pathological examination and the visual lameness scoring of the lame animals are shown in Table 1.

**Table 1:** Gender, weight, diagnosis and localization of lameness in lame pigs

**ID no.**	**Gender**	**Body mass (kg)**	**Lame limb**	**Diagnosis**	**Location**	**Visual lameness score**
2456	F	10.3	RF	Periarthritis	Carpus	2
5685	M	13	RH	Periarthritis + Arthritis	Tarsus	4
4155	M	7.4	LH	Arthritis	Metatarsophalangeal joint	3
2602	M	10.7	RH	Periarthritis	Distal interphalangeal joint	2
6744	M	8.6	LH	Periarthritis	Tarsus + knee	2
9559	F	7.8	RF	Periarthritis + Arthritis	Metacarpophalangeal joint	3
7173	M	8.5	RF	Periarthritis	Elbow	4
2879	F	9.7	RH	Periarthritis + Arthritis	Tarsus	3
2408	F	12.1	LH	Arthritis	Metatarsophalangeal joint	4
4751	M	7.2	LF	Periarthritis	Carpus	3

**Figure 1:**
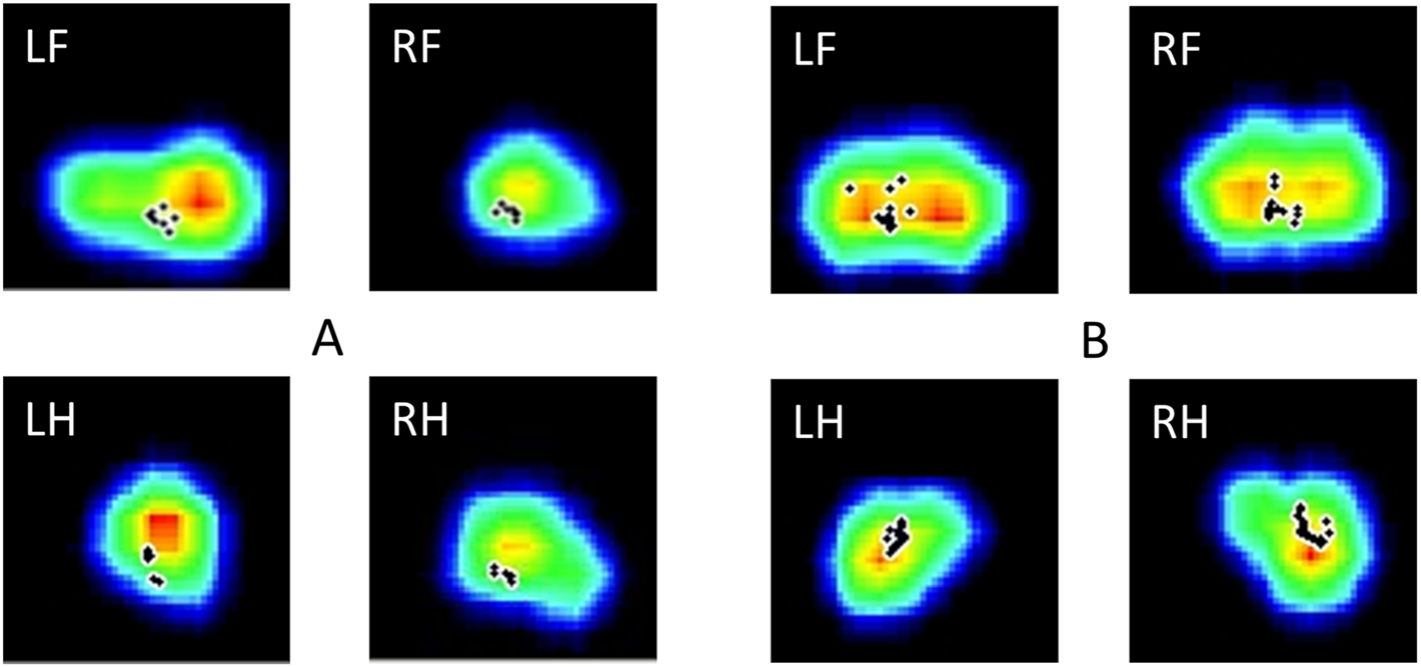
**Typical pressure mat recordings from pig with right forelimb lameness (A) and sound pig (B).** LF = left front, RF = right front, LH = left hind, RH = right hind. Pig A had a visual lameness score of 2/5. The recorded pressure is represented by color: red for the highest pressure, blue for the lowest pressure. Black dots represent the center of pressure throughout the stance phase. Pig A is showing a clear difference in the amount of pressure applied between the lame foot (RF) and the contralateral foot (LF), while also showing a pressure redistribution toward the left hind limb.

Pressure mat analysis yielded prints for each claw (See Figure 1 for an example of pressure mat recordings).

### Redistribution of pressure

**Figure 2:**
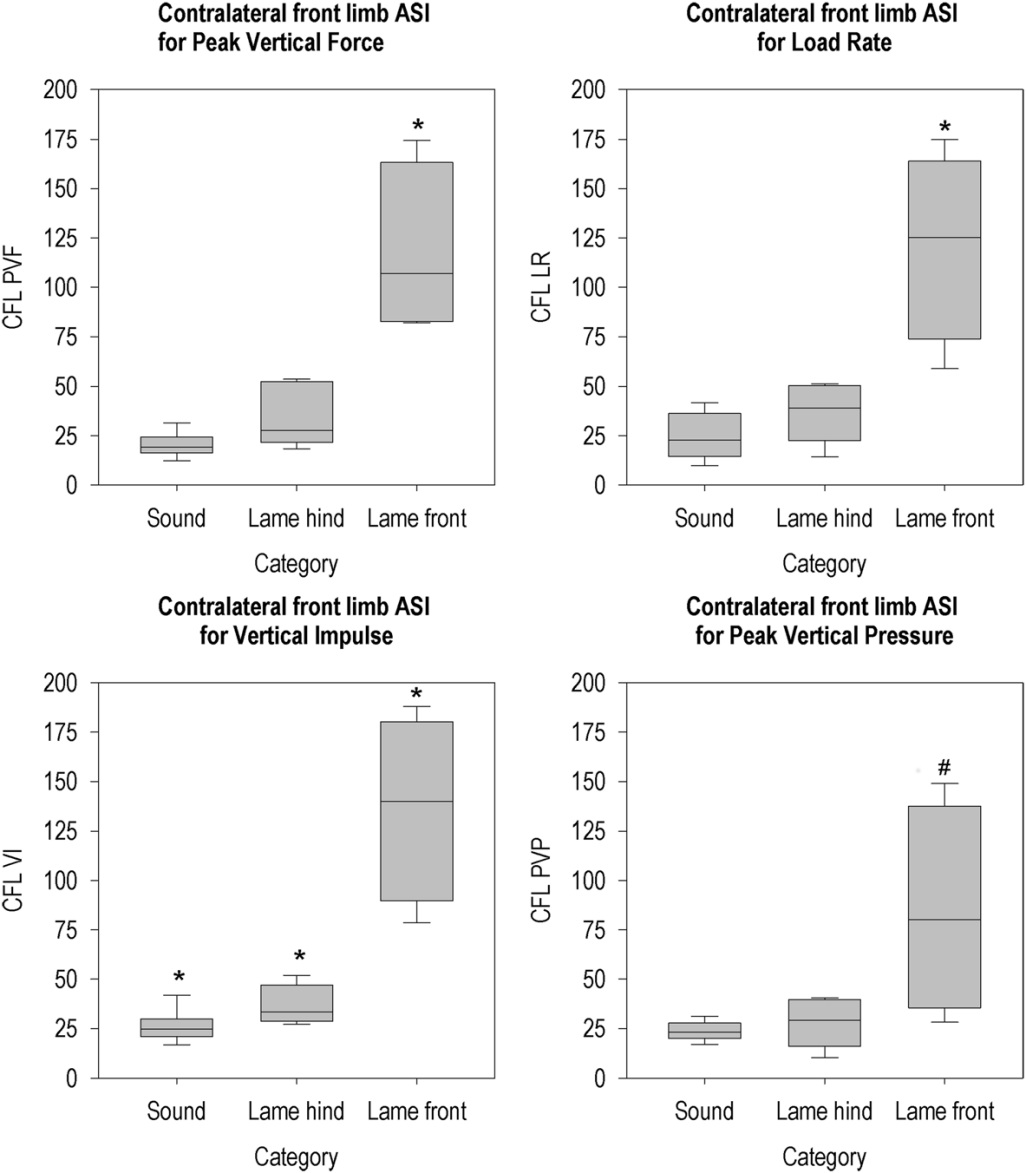
**Contralateral forelimb ASI per group for all parameters.** *The ASI of this group differs from the ASIs of the other two groups (p < 0.05). # The ASI of this group differs from the sound group (p < 0.05).

**Figure 3:**
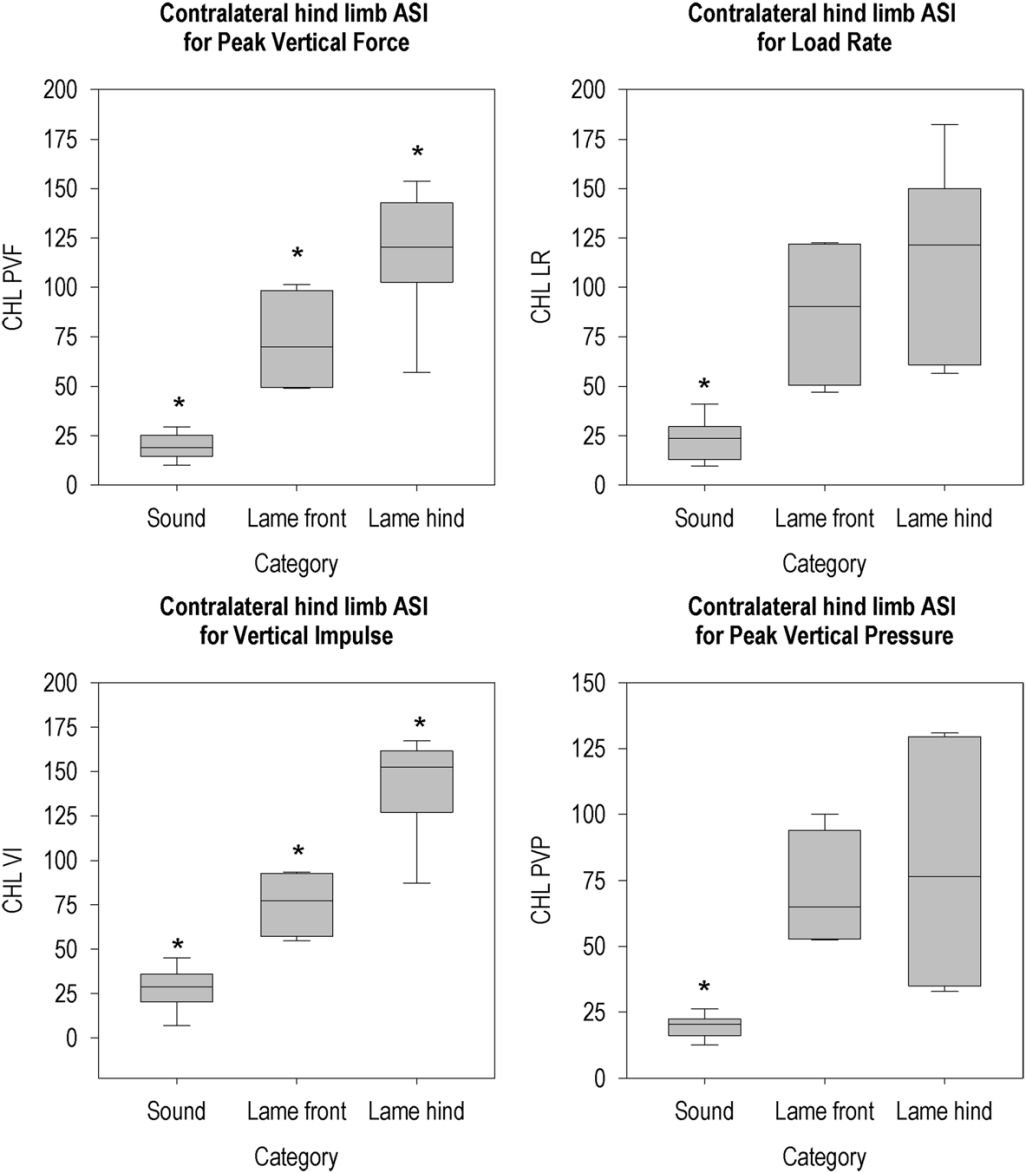
**Contralateral hind limb ASI per group for all parameters.** *The ASI of this group differs from the ASI’s of the other two groups (P < 0.05).

**Figure 4:**
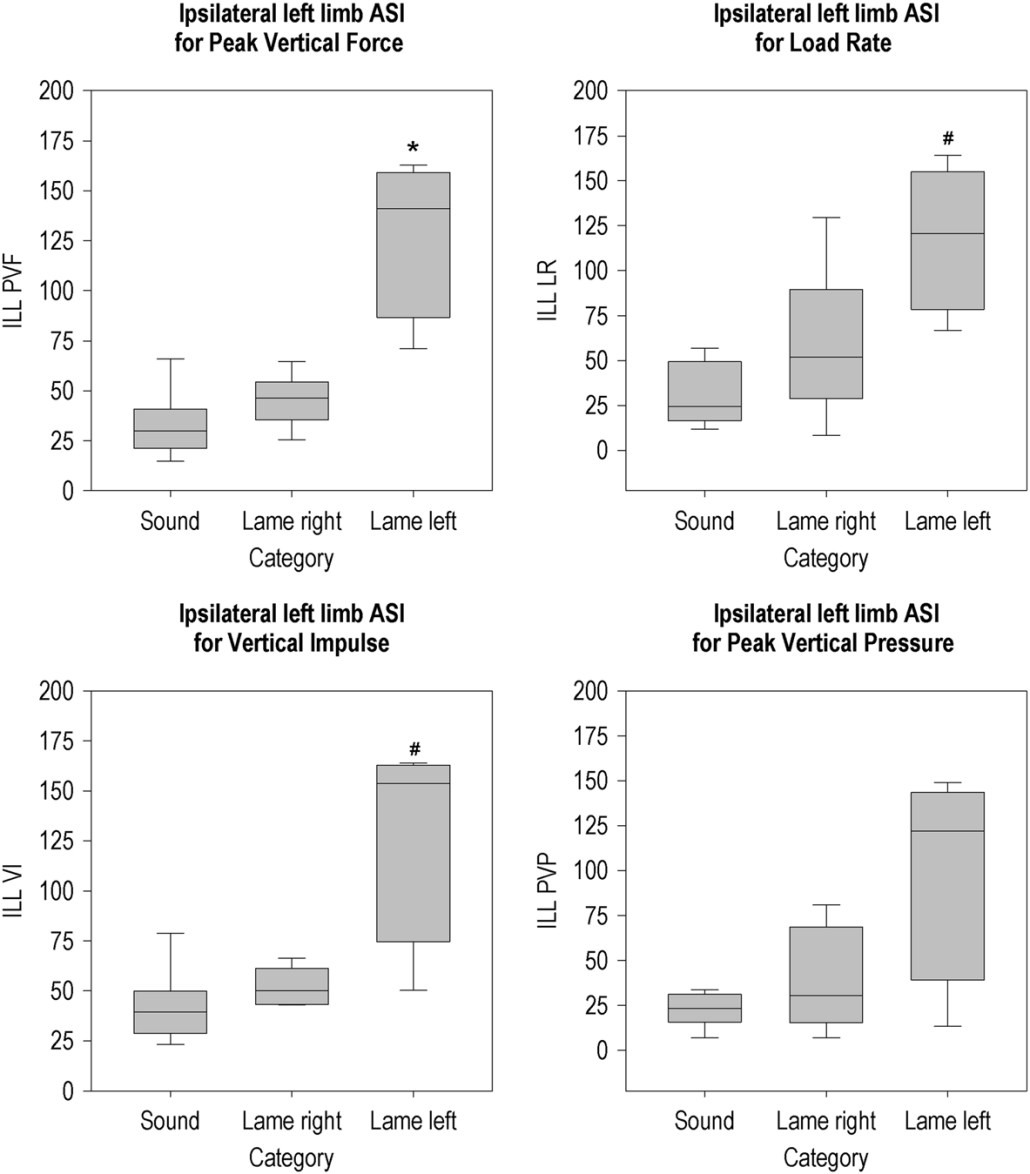
**Ipsilateral left limb ASI per group for all parameters.** *The ASI of this group differs from the ASIs of the other two groups (P < 0.05). # The ASI of this group differs from the sound group (P < 0.05).

**Figure 5:**
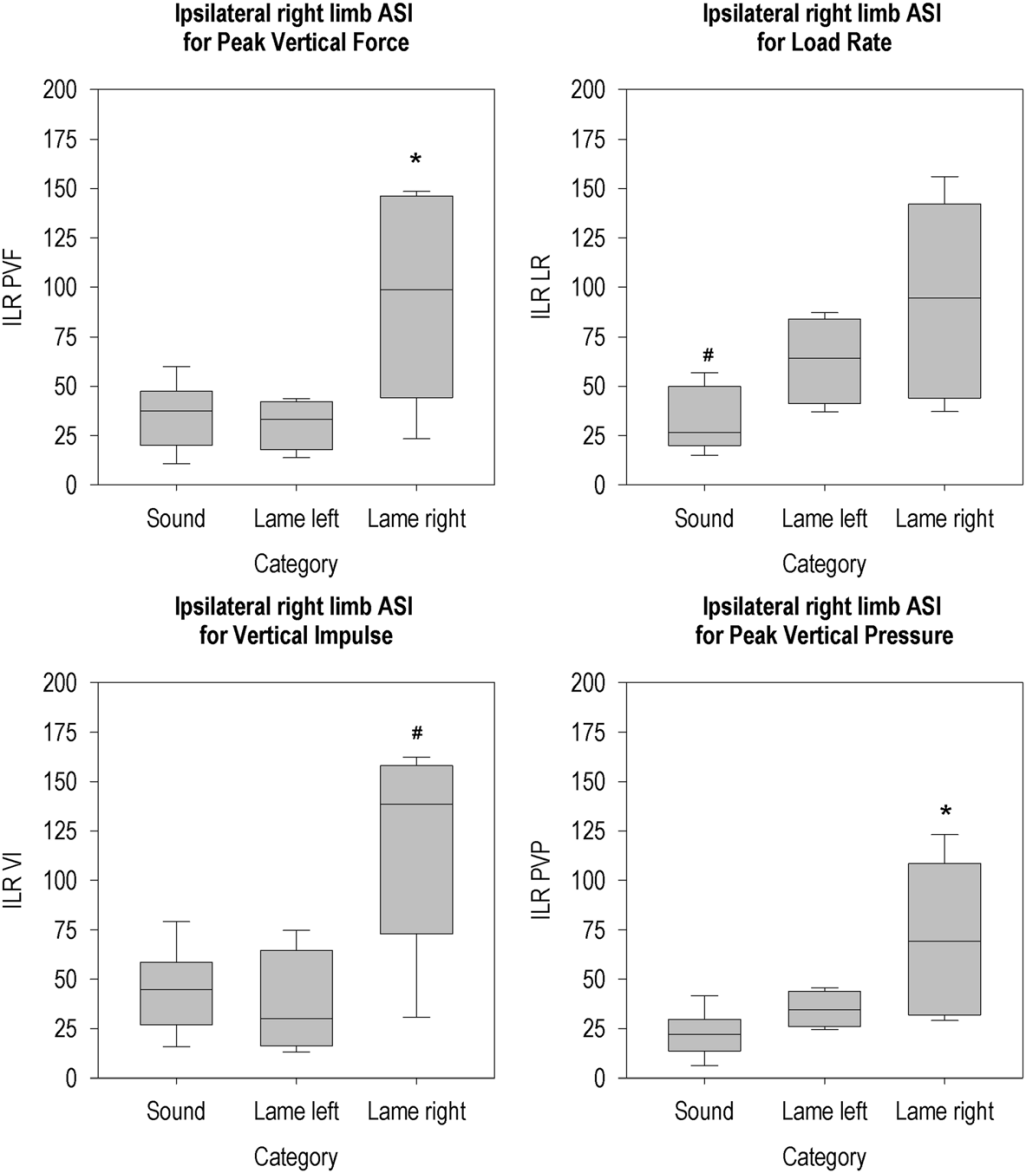
**Ipsilateral right limb ASI per group for all parameters.** *The ASI of this group differs from the sound group (P < 0.05). # The ASI of this group differs from the ASIs of the other two groups (P < 0.05).

**Figure 6:**
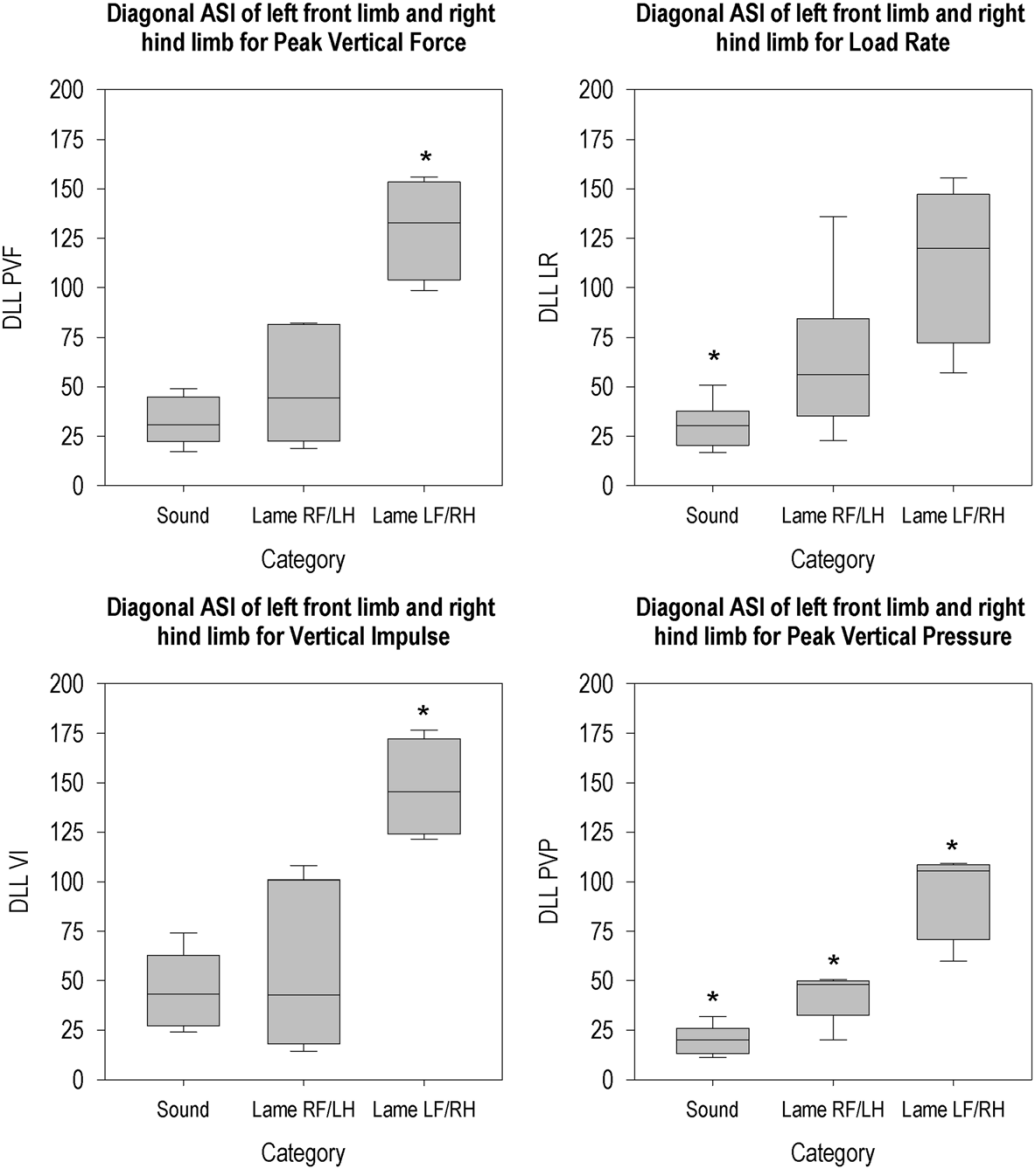
**Diagonal ASI for left forelimb and right hind limb per group for all parameters.** *The ASI of this group differs from the ASIs of the other two groups (P < 0.05).

**Figure 7:**
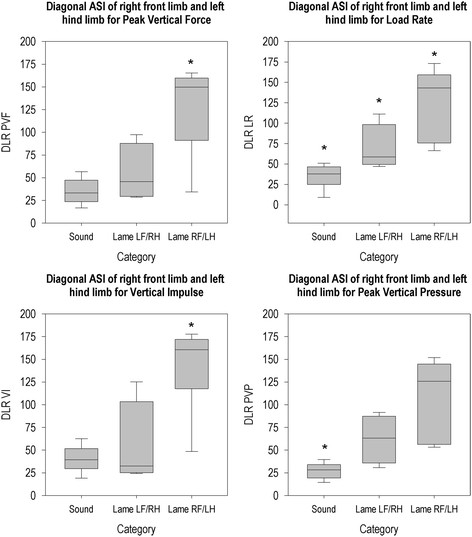
**Diagonal ASI for right front limb and left hind limb per group for all parameters.** *The ASI of this group differs from the ASIs of the other two groups (P < 0.05).

Figures 2, 3, 4, 5, 6 and 7 present the ASIs of three groups of animals: sound animals, animals that were lame on a limb that was assessed by that particular ASI and animals that were lame on a limb that was not assessed by that ASI. Full test statistics and exact p-values are provided as Additional file 1.

Contralateral forelimb ASI for PVF differed between groups (χ^2^(2, N = 20) = 11.61, p = 0.003) and was higher in the animals that were lame on a forelimb compared to animals that were lame on a hind limb or animals that were not lame (see Figure 2). Load rate CFL differed between groups as well (χ^2^(2, N = 20) = 11.09, p = 0.004) and animals that were lame on a forelimb had higher CFL than animals that were lame on a hind limb and sound animals. A difference between all groups was present in the CFL of VI (χ^2^(2, N = 20) = 12.19, p = 0.002). Although CFL of PVP also differed between groups (χ^2^(2, N = 20) = 7.01, p = 0.030), the Mann-Whitney U test only showed a difference between sound animals and animals that were lame on a forelimb.

Contralateral hind limb ASI (Figure 3) was different between groups for all parameters (PVF (χ2(2, N = 20) = 15.48, p = 0.000), LR (χ2(2, N = 20) = 14.58, p = 0.001), VI (χ2(2, N = 20) = 15.48, p = 0.000) and PVP (χ2(2, N = 20) = 14.29, p = 0.001)). The posthoc Mann-Whitney U test showed a difference between all groups for PVF and VI. Sound animals had lower ASIs than animals that were lame on a forelimb or animals that were lame on a hind limb for LR and PVP, but there was no difference between animals that were lame on a forelimb and animals that were lame on a hind limb for these parameters.

The ASI of the ipsilateral left limbs (Figure 4) differed between the three groups for PVF (χ^2^(2, N = 20) = 11.09, p = 0.004), LR (χ^2^(2, N = 20) = 9.07, p = 0.011) and VI (χ2(2, N = 20) = 8.73, p = 0.013), but not for PVP. In the post-hoc analysis of ILL of PVF, pigs that were lame on the left limb had higher ILLs than pigs that were lame on the right limb and sound pigs. ILL of LR and VI were higher in pigs that were lame on the left limb compared to sound animals.

Ipsilateral right limb ASI (Figure 5) differed between groups for PVF (χ^2^(2, N = 20) = 6.32, p = 0.042) and PVP (χ^2^(2, N = 20) = 9.53, p = 0.009), and the posthoc Mann-Whitney U test showed the animals that were lame on a right limb had higher ASIs than sound animals. The difference between groups was also found for LR (χ^2^(2, N = 20) = 8.22, p = 0.016), with sound animals having lower ASIs than lame animals, regardless of which limb was lame. VI (χ^2^(2, N = 20) = 7.84, p = 0.020) differed between groups with the animals that were lame on the right limbs having higher ASIs than other animals.

For all parameters, diagonal LF/RH ASI (Figure 6) was different between groups (PVF (χ^2^(2, N = 20) = 9.52, p = 0.009), LR (χ^2^(2, N = 20) = 10.94, p = 0.004), VI (χ^2^(2, N = 20) = 9.15, p = 0.010), PVP (χ^2^(2, N = 20) = 13.91, p = 0.001)). Animals that were lame on the left fore or right hind limb had higher DLL than animals than animals that were lame on the right fore or left hind limb and had higher DLL than sound animals for PVF and VI. LR DLL was lower in sound animals than in other animals. For PVP, all groups differed from each other, with animals that were lame on the left fore or right hind limb having higher DLL than animals that were lame on the right fore or left hind limb and sound animals.

Diagonal ASI of right front limb and left hind limb (Figure 7) differed between groups for all parameters (PVF (χ^2^(2, N = 20) = 9.14, p = 0.010), LR (χ^2^(2, N = 20) = 14.66, p = 0.001), VI (χ^2^(2, N = 20) = 9.33, p = 0.009), PVP (χ^2^(2, N = 20) = 13.01, p = 0.001)). Pigs that were lame on the right fore or left hind limb had higher DLR for PVF and VI compared to the sound group. For LR, all groups differed from each other. Sound animals had lower PVP DLR than lame animals.

**Figure 8:**
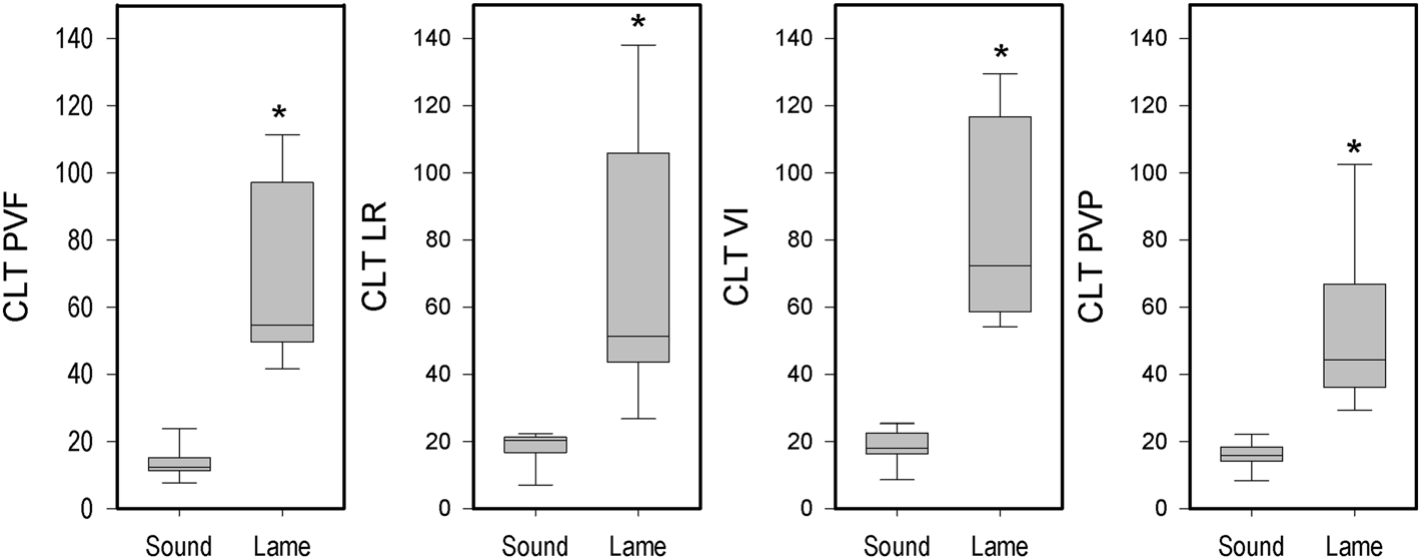
**Boxplots for CLT for PVF, LR, VI and PVP for the lame and sound groups.** *This group differs from the sound group (P < 0.05).

CLT (Figure 8) was higher in lame animals compared to sound animals for all parameters (PVF: t(18) = -6.11, p = 0.000, LR: t(18) = -4.22, p = 0.002), VI: t(18) = -6.80, p = 0.000, PVP: t(18) = -4.60, p = 0.001).

### Correlation with visual scoring

The visual score of the 20 pigs correlated highly with CLT of PVF (r = 0.82, p < 0.05), CLT of VI (r = 0.83, p < 0.05), CLT of LR (r = 0.80, p < 0.05) and CLT of PVP (r = 0.77, p < 0.05).

### ROC analysis

The ROC analysis showed distinct cutoff values with 100% sensitivity and specificity for all four parameters. The cutoff value calculated from the ROC analysis was 33.1 for CLT of PVF, 24.0 for CLT of LR, 39.8 for CLT of VI and 25.6 for CLT of PVP.

## Discussion

Data collection from both lame and sound pigs appeared relatively simple, as both groups showed exploratory behavior and a strong response to the treats. Lame pigs walked at a slower mean velocity than sound pigs. This is in agreement with findings in sows [14] and cattle [15],[16]. Velocity itself may provide an indicator for lameness. However, there is some overlap between lame pigs and sound pigs on this measure. Also, in practice, velocities can only be measured and compared under standardized conditions. It may be difficult to distinguish the different velocities by visual judgment. Lastly, velocity per se does not provide any clue as to which limb is the lame one. Directional ASI’s can provide this information. Velocity, however, may be a first indicator to direct a farmer’s or veterinarian’s attention to certain pigs; a pig moving exceptionally slow may warrant further examination. Incorporation of “walking speed” as a parameter into lameness scoring protocols therefor may be advisable.

The lame pigs in this study were selected from a commercial breeding farm without prior knowledge of the cause of the lameness. As a result, the group with the lame pigs was rather heterogeneous, with several pathomorphological diagnoses on different locations in the limbs. Since the kinetic parameters we used were representing the forces in the entire limb, we did not expect location of the lesion (proximal or distal in the limb) to influence the magnitude of ASIs. However, the study group was not large enough to make any definitive statements on this subject.

### Redistribution of pressure mat parameters

#### Contralateral

The contralateral asymmetry was assessed from the CFL and CHL parameters. CFL compares the two forelimbs to each other. If a pig is lame on a forelimb, it will reduce the amount of force put on that limb by shifting it towards the other forelimb, and to a lesser extent to the ipsilateral and diagonal hind limbs. Thus, it is possible that in a pig that is lame on a forelimb, the hind limb asymmetry (CHL) also increases due to a mechanical compensation, as there is no pain related lameness in these hind limbs. In our experiment, as expected the CFL was increased in forelimb lame pigs compared to that recorded in sound pigs. The CHL of pigs that were lame on a forelimb was also higher than that of the sound animals. The increased asymmetry in the forelimbs was also found in studies on lame horses [17] and dogs [18],[19] for PVF and VI, and for PVP in lame pigs [13]. In both the aforementioned equine and canine studies, the increased asymmetry was mainly due to a significant decrease in PVF and VI in the lame limb. The PVF and VI in the contralateral limb did not increase significantly in lame dogs at walk, but did increase in horses at higher lameness grades.

In agreement with our study, also in forelimb lame dogs and horses the CHL similarly increased for both PVF as well as VI. This may be due to a significant force shift from the lame forelimb to the diagonal hind limb. Moreover, this has been attributed to unloading of the limb ipsilateral to the lame limb (i.e., when RF is lame RH is also unloaded) [20]. We also saw a shift towards the diagonal limb in our lame animals, which would cause asymmetry due to a mechanical lameness also in the hind limbs, even though there is no true pain related lameness present in these limbs.

The load distribution between the two hind limbs is quantified by the CHL. Indeed, the CLH of the hind limb lame pigs in our experiment increased, similar as to earlier findings in hind limb lame dogs, horses and pigs [10],[11],[13],[21],[22]. The increased asymmetry may be due to a decreased loading of the lame limb, which was found in horses [22] and dogs [21] and additionally due to an increased loading of the contralateral hind limb. The latter mechanism was observed in the study by Fischer et al. [21], whereas in the study by Weishaupt et al. [22] it was only observed for VI and not for PVF.

The CFL of animals that were lame on a hind limb was significantly higher than that of sound animals only for VI. This may be because the lameness mainly affects stance time rather that the PVF. This adaptation of timing rather than loading may be a more subtle manifestation of compensation in lame pigs. A significant increase of stance time was found using kinematics in lame sows [14]. An increased CFL in walking dogs that were lame on a hind limb was found both for PVF and VI [21], but in trotting horses that were lame on a hind limb no increased asymmetry was found [22]. In the dogs, the ipsilateral forelimb had an increased PVF and the diagonal forelimb had an increased VI, thereby influencing forelimb symmetry.

#### Ipsilateral

The fore-hind symmetry is assessed by the ILL and ILR. Generally, we expected that front-hind asymmetry would increase on the side of the lesion, as was shown previously in dogs [18] and pigs [13]. Again, the main reason for this asymmetry was unloading of the lame limb [17]–[19],[21],[22]. With the exception of the study by Fischer [21], who found an increased PVF in the ipsilateral limb of dogs, no increase in loading of the ipsilateral limb was found in these studies. In our experiment, we also found an increase in asymmetry on the ipsilateral side of the lameness with the exception of ILL of PVP. It may be that the same mechanism that cause lameness in the forelimbs also caused contralateral asymmetry in the hind limbs, namely unloading of the ipsilateral limb [20], causes the difference between the ipsilateral limbs to become smaller, resulting in an increase in asymmetry that is not statistically significant.

There was no increase in ipsilateral symmetry on the side opposite the lesion, except for LR. This is in contrast with the study on walking dogs [18], where a significant increase in ipsilateral asymmetry of PVF and VI was seen on the sound side as well as on the lame side. This change only in load rate may represent a subtle manifestation of compensation.

#### Diagonal

Lameness in the left fore or right hind limb caused an increase in DLL. Lameness in the right fore or left hind limb caused an increase in DLR. This increase was expected, as several studies on horses and dogs have shown redistribution of force away from the lame limb and towards the diagonal limb [17]–[19]. Lameness on a diagonal limb pair, however, did not always influence the other diagonal. The only observable change in ASI of diagonal limbs in pigs that were lame on the limbs outside the diagonal limb pair, was seen for LR and PVP. Bockstahler et al. [18] did not find a similar significant change in diagonal ASIs for the non-lame limbs for PVF and VI, like was found in our study. This may be due to the relatively high degree of lameness of the pigs in our study, which may have caused a larger effect on other limbs compared to the study by Bockstahler et al.

### Correlation with visual scoring

Correlations between visual scoring of lameness and CLTs of all parameters were high. This finding is in agreement with the study of Oosterlinck et al. in dogs [11], who also found good correlations between visual scoring and PVF, VI, and PVP, as recorded using a pressure mat. In that study, PVP was the parameter that correlated lowest with visual gait scores, in contrast to the findings in our study. Oosterlinck et al. [11] hypothesized that the decrease in limb loading of the lame limb combined with a concurrent decrease in contact area resulted in a “falsely” low pressure value (which is force per unit of area), rendering PVP as a less reliable parameter. A study in horses [23] that compared visual lameness scoring to force plate, also found significant correlations, particularly with PVF and VI. Quinn et al. [24], however, did not find correlations between PVF measured by force plate and numerical rating scale scores of three observers scoring lame dogs. Only one out of three observers had a significant correlation between the numerical rating scale score and VI as recorded using a force plate. In that study, the highest correlation was only found when dogs were considerably lame. Our study did not include pigs that had a lameness score of 1 (subtle lameness. This may be a reason for the discrepancy between our findings and those of Quinn et al. [24]. It is also possible that ASIs of kinetic parameters provide better correlation with visual scoring than absolute values, as a visual evaluation is an interpretation of symmetry of locomotion rather than a measure of absolute limb loading. Often, compensatory load redistribution to the contralateral limb occurs, thus enhancing asymmetrical limb kinetics and kinematics [17],[22],[25].

### ROC analysis

In the present study, we assessed the use of CLT of a set of only four pressure mat parameters to distinguish lame from sound pigs. Lameness has often been described in terms of asymmetry. A previous study by Karriker et al. [13] found a substantial decrease in symmetry of PVP in an experimental lameness model in sows. However, no cutoff values were estimated yet for these ASIs to distinguish lame pigs from sound pigs. In dogs, however, these cutoff values already have been established. Fanchon et al. [10] used an instrumented treadmill to compare ASIs for PVF, VI and LR and found PVF to be the only ASI with a high accuracy. Oosterlinck et al. [11] found ASIs of PVF, VI and contact area to be highly accurate, in contrast to the ASI of PVP. In both studies, it was known that the dogs were lame on the hind limbs. However, in a clinical setting it may not always be clear which is the lame limb. In our study, the pigs were lame only on one of the four limbs. Therefore, we used the CLT as a potential tool to determine the presence of lameness.

A certain degree of asymmetry was present in sound animals as well as in lame animals. No animal was completely symmetrical, which is in agreement with findings in dogs [26] and horses [5]. Using ROC analysis, optimal cutoff points were found for all four overall left-right ASIs, with a 100% sensitivity and specificity. In future studies, it would thus be interesting to assess the performance of the pressure mat as a tool to detect very subtle lameness. In this study, there were no pigs with a lameness score of 1 (very subtle lameness) used, as such pigs are not easily identified on a farm in a practical situation.

## Conclusion

In conclusion, lameness in walking pigs causes an increase in contralateral, ipsilateral and diagonal asymmetry, due to unloading of the lame limb combined with redistribution of this load to the non-lame limbs. However, the six ASI’s that were studied only took into account two limbs at a time. Animals that were lame on a limb that was not assessed by that ASI did not always have a higher asymmetry than sound animals. In situations where it is unknown which is the lame limb, a method that takes into account all four limbs is needed. An example of such a method is the use of overall left-right ASIs.

Using the overall left-right ASIs we were able to distinguish lame pigs from sound pigs with 100% sensitivity and specificity, which correlated well with subjective lameness scores. They may provide a future objective method to assess lameness and the effect of interventions on the presence of lameness.

## Methods

The study was reviewed and approved by the local ethical committee of Utrecht University (DEC no 2012.III.05.04), The Netherlands, and was conducted in accordance with the recommendations of the EU directive 86/609/EEC. All effort was taken to minimize the number of animals used and their suffering.

### Animals

A group of n = 10 clinically lame and of n = 10 sound Topigs 20 × Tempo pigs (6 male, 4 female per group) were selected by a veterinarian at a commercial breeding farm. Only pigs that were clinically lame on one limb, that could stand and walk unaided and that did not show signs of any other disease were selected. The group of sound pigs was assessed as one total batch, while due to logistics the lame pigs were evaluated in 3 batches 2 weeks apart. All pigs were clinically examined at the farm by a veterinarian to make sure they fitted the inclusion criteria for this study, before they were transported to the research facility of the Veterinary Faculty, Department of Farm Animal Health, Utrecht University.

### Housing

The pigs were housed at the research facility of Utrecht University. They were kept in small subgroups of 3-5 pigs in pens with closed concrete floors measuring 153 cm × 256 cm that were covered with sawdust. The pigs were provided with 11 hours of light per day (from 7 a.m. to 6 p.m.) from both daylight and artificial lighting. They were housed in a stall with an ambient temperature between 22 and 24°C and one extra heatlamp per pen was provided. The animals had ad libitum access to food (Groeiporco, De Heus Animal Nutrition, Ede, The Netherlands) and water, and were provided with enrichment toys (plastic ball, metal chain) during the entire experiment.

### Data recording

Upon arrival at the research facility, the lame pigs (first batch n = 4 pigs, second batch n = 3 pigs, third batch n = 3 pigs) were put together in one single pen, without any sound animals in the same pen. The sound pigs were grouped according to size in two subgroups of 5 pigs. All pigs were allowed to acclimatize for one day before entering the experiment. At the starting day of the experiment, piglets were weighed and clinically examined (breathing rate, heart rate, rectal temperature, assessment of skin, mucous membranes and lymph nodes).

The pressure mat recordings were performed using a Footscan® 3D Gait Scientific 2 m system (RSscan International, Olen, Belgium) with an active sensor surface of 1.95 m × 0.32 m containing 16384 sensors (2.6 sensors per cm^2^), with a sensitivity of 0.27-127 n/cm^2^ and a measuring frequency of 126 Hz. The pressure mat was connected to a laptop with dedicated software (Footscan Scientific Gait 7 gait 2^nd^ generation, RSscan International, Olen, Belgium). The mat was placed in a custom-built runway as used by Meijer et al. [27]. The pressure mat was calibrated according to the manufacturer’s instructions by a person weighing approximately 70 kg.

Visual scoring was performed according to the method described by Main et al. [28]. This scoring system yields a score from 0 to 5, with 0 being a sound individual and 5 an extremely lame pig. Observations were first made in the home pen of the piglets without disturbing them. Posture, behavior and gait were marked. After that, the observer approached the pigs and opened the pen to note the behavioral response to this stimulus. Finally, if pigs had not risen yet, the observer encouraged the pig to stand up so that locomotion could be scored.

For the pressure mat analysis, one pig at a time was let out of its pen and was allowed to walk freely to the holding area. Very lame pigs that were reluctant to leave the pen were carried. Once inside the holding area, the pig was allowed to acclimatize for one minute. After this, the door to the runway was opened and the pig could walk to the holding area at the other side. All pigs eventually started to explore their surroundings and crossed the runway. Every time they did, they were rewarded with candy. Exploratory behavior together with rewards was sufficient to collect three correct runs per pig. A run was considered correct when the pig walked (gait confirmed by duty factor) across the runway without stopping at a steady velocity and looking straight ahead.

After all data had been collected, the pigs were euthanized. They were sedated using a 2 mg/kg intramuscular injection of Azaperone (Stresnil, Elanco Animal Health, Greenfield, USA) and subsequently euthanized by intracardial injection of 200 mg/kg Pentobarbital (Euthanimal, Alfasan, Woerden, The Netherlands). Gross pathology was performed at the Department of Pathobiology of the Faculty of Veterinary Medicine of Utrecht University, with specific attention paid to their limbs, and in particular the lame limb.

### Data analysis

The collected footprints from their claws in the three runs were manually assigned to left fore (LF), right fore (RF), left hind (LH) and right hind (RH) limb using the software provided by the pressure plate manufacturer. PVF (N), LR (N/s), VI (Ns) and PVP (N/cm^2^) were normalized for body mass.

**Figure 9:**
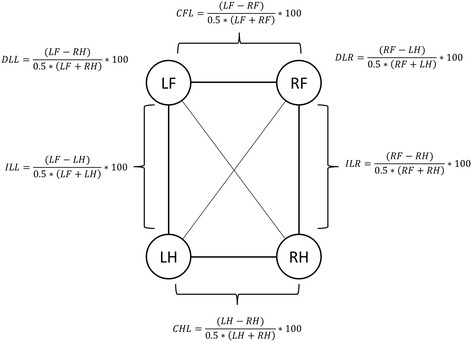
**Formulas used for calculation of the various ASIs.** CFL: contralateral forelimbs, CHL = contralateral hind limbs, ILL = ipsilateral left limbs, ILR = ipsilateral right limbs, DLL = diagonal left fore and right hind limbs, DLR = diagonal right fore and left hind limbs. LF = left fore, RF = right fore, LH = left hind, RH = right hind.

ASIs were calculated for each variable using modifications of the formula introduced by Oomen et al. [29] Figure 9.

The total left-right ASI (CLT) compared the left limbs to the right limbs and was calculated using the formula:

(1) $$\mathrm{CLT}=\frac{\left(\mathrm{LF}+\mathrm{LH}\right)-\left(\mathrm{RF}+\mathrm{RH}\right)}{0.5*\left(\left(\mathrm{LF}+\mathrm{LH}\right)+\left(\mathrm{RF}+\mathrm{RH}\right)\right)}*100$$

These formula’s for ASIs yield a score between -200 and + 200. Both extreme values indicate very severe (non-weight bearing) lameness. The direction of the extreme (negative or positive) indicated the direction of the weight redistribution. An ASI of 0 indicates perfect symmetry.

To determine redistribution, the ability of ASIs to identify lame pigs, and the correlation between visual scoring and ASIs, the absolute value of the ASIs was used, removing the distinction between right- or left-sided asymmetry. This yields a score between 0 and 200, with higher values indicating relatively higher loading of the left or right limb and 0 indicating perfect symmetry. Mean ASIs were calculated from the 3 ASIs per pig.

### Statistics

The body mass, velocity and CLT of the lame and sound groups were compared using an independent-samples *t*-test. The data for body mass and velocity had equal variances in both groups according to Levene’s test, but the data from the CLT did not, therefore the non-parametric Mann-Whitney U test was used.

To assess whether redistribution was taking place, the animals were divided into 3 groups for the six ASIs comparing two limbs (CFL, CHL, ILL, ILR, DLL, DLR): sound animals, animals that were lame on a limb that was assessed by that particular ASI and animals that were lame on a limb that was not assessed by that ASI. For example, for the CFL there were three groups: sound animals, animals that were lame on the left front limb or the right front limb (the limbs that are assessed by CFL) and animals that were lame on the left hind limb or the right hind limb (the limbs that are not assessed by the CFL). Since the data were not distributed normally (confirmed by Kolmogorov-Smirnov test), a Kruskall-Wallis test was used to compare the three groups. A Mann-Whitney test with Bonferroni correction was performed as a post-hoc test to assess the differences between groups.

Spearman’s rank correlation coefficient was used to evaluate correlations between visual scores and CLT’s.

Receiver-operated curve analysis [30] was performed to assess the performance of each of the CLT’s as diagnostic test in the diagnosis of lameness. The sensitivity (y axis) was plotted against 1-specificity (x-axis) for each possible cutoff value. A diagonal line where sensitivity is equal to 1- specificity represents a discriminating ability of the test that is no better than chance. The top left corner represents 100% sensitivity and specificity. The resulting area under the curve (AUC) was used to assess the performance of each of the CLT’s.

All data are presented as means ± SD. Statistical significance was set at *p <* 0.05.

## Abbreviations

LF: Left forelimb

RF: Right forelimb

LH: Left hind limb

RH: Right hind limb

PVF: Peak Vertical Force

LR: Load Rate

VI: Vertical Impulse

PVP: Peak Vertical Pressure

ASI: Asymmetry index

CLT: Asymmetry of both left limbs (LF and LH) vs. both right limbs (RF and RH)

CFL: Asymmetry of contralateral forelimbs

CHL: Asymmetry of contralateral hind limbs

ILL: Asymmetry of left ipsilateral limbs

ILR: Asymmetry of right ipsilateral limbs

DLL: Asymmetry of diagonal fore- and hindlimb (LF vs. RH)

DLR: Asymmetry of diagonal fore- and hindlimb (RF vs. LH)

## Competing interests

The authors declare that they have no competing interests.

## Authors’ contributions

EM contributed to study design and data collection, analyzed the data and drafted the manuscript. MO provided advice on pressure mat data collection and analysis, and critically revised the manuscript. AvN critically revised the manuscript. WB contributed to study design, provided advice on automated gait analysis and critically revised the manuscript. FJS aided in writing the first draft of the manuscript, provided advice on data analysis and critically revised the manuscript. All authors read and approved the final manuscript.

## Additional file

## Supplementary Material

Additional file 1: Full test statistics and exact p-values for all ASI's. Click here for file
