# Supplementary material for: Pressure mat analysis of naturally occurring lameness in young pigs after weaning
Source: BMC Vet Res. 2014 Aug 20;10:193. doi: 10.1186/s12917-014-0193-8 (PMC4236822; doi:10.1186/s12917-014-0193-8)
Supplement: Additional file 1: — Full test statistics and exact p-values for all ASI's. [file s12917-014-0193-8-S1.pdf]

Full test statistics and exact p – values for all ASI's

Contralateral					
**Variable**	**KW chi-square**	***P*-value**	**Group**	**Mann-Whitney *U***	***P*-value**
CFL PVF	11.61	0.003	Sound – lame front	40	0.006
			Sound – lame hind	48	0.058
			Lame front – lame hind	24	0.014
CFL LR	11.09	0.004	Sound – lame front	40	0.006
			Sound – lame hind	46	0.093
			Lame front – lame hind	24	0.014
CFL VI	12.19	0.002	Sound – lame front	40	0.006
			Sound – lame hind	50	0.034
			Lame front – lame hind	24	0.014
CFL PVP	7.01	0.030	Sound – lame front	38	0.013
			Sound – lame hind	37	0.481
			Lame front – lame hind	21	0.070
CHL PVF	15.48	0.000	Sound – lame hind	60	0.001
			Sound – lame front	40	0.006
			Lame front – lame hind	2	0.043
CHL LR	14.58	0.001	Sound – lame hind	60	0.001
			Sound – lame front	40	0.006
			Lame front – lame hind	7	0.337
CHL VI	15.48	0.000	Sound – lame hind	60	0.001
			Sound – lame front	40	0.006
			Lame front – lame hind	2	0.043
CHL PVP	14.29	0.001	Sound – lame hind	60	0.001
			Sound – lame front	40	0.006
			Lame front – lame hind	12	1.000

Ipsilateral					
**Variable**	**KW chi-square**	***P*-value**	**Group**	**Mann-Whitney *U***	***P*-value**
ILL PVF	11.09	0.004	Sound – lame left	40	0.006
			Sound – lame right	46	0.093
			Lame left – lame right	24	0.014
ILL LR	9.07	0.011	Sound – lame left	40	0.006
			Sound – lame right	44	0.143
			Lame left – lame right	20	0.110
ILL VI	8.74	0.013	Sound – lame left	38	0.013
			Sound – lame right	46	0.093
			Lame left – lame right	21	0.070
ILL PVP	3.86	0.145	Sound – lame left	32	0.104
			Sound – lame right	39	0.357
			Lame left – lame right	19	0.166
ILR PVF	6.32	0.042	Sound – lame right	51	0.026
			Sound – lame left	16	0.621
			Lame left – lame right	3	0.070
ILR LR	8.22	0.016	Sound – lame right	52	0.020
			Sound – lame left	35	0.040
			Lame left – lame right	6	0.241
ILR VI	7.84	0.020	Sound – lame right	53	0.015
			Sound – lame left	14	0.437
			Lame left – lame right	2	0.043
ILR PVP	9.53	0.009	Sound – lame right	56.5	0.005
			Sound – lame left	32	0.104
			Lame left – lame right	6	0.241

Diagonal					
Variable	KW chi-square	*P*-value	Group	Mann-Whitney *U*	*P*-value
DL PVF	9.52	0.009	Sound – Lame left front or right hind	40	0.006
			Sound – Lame right front or left hind	37	0.481
			Lame left front or right hind – lame right front or left hind	24	0.014
DLL LR	10.94	0.004	Sound – Lame left front or right hind	40	0.006
			Sound – Lame right front or left hind	51	0.026
			Lame left front or right hind – lame right front or left hind	19	0.166
DLL VI	9.15	0.010	Sound – Lame left front or right hind	40	0.006
			Sound – Lame right front or left hind	29	0.957
			Lame left front or right hind – lame right front or left hind	24	0.014
DLL PVP	13.90	0.001	Sound – Lame left front or right hind	40	0.006
			Sound – Lame right front or left hind	55	0.008
			Lame left front or right hind – lame right front or left hind	24	0.014
DLR PVF	9.14	0.010	Sound – Lame right front or left hind	55	0.008
			Sound – Lame left front or right hind	29	0.229
			Lame left front or right hind – lame right front or left hind	2	0.043
DLR LR	14.66	0.001	Sound – Lame right front or left hind	60	0.001
			Sound – Lame left front or right hind	38	0.013
			Lame left front or right hind – lame right front or left hind	2	0.043
DLR VI	9.33	0.009	Sound – Lame right front or left hind	56	0.006
			Sound – Lame left front or right hind	19	0.944
			Lame left front or right hind – lame right front or left hind	1	0.025
DLR PVP	13.01	0.001	Sound – Lame right front or left hind	60	0.001
			Sound – Lame left front or right hind	35	0.040
			Lame left front or right hind – lame right front or left hind	4	0.110
